# Supplementary material for: Electrocaloric cooling system utilizing latent heat transfer for high power density
Source: Commun Eng. 2024 Mar 21;3:55. doi: 10.1038/s44172-024-00199-z (PMC10957880; doi:10.1038/s44172-024-00199-z)
Supplement: Supplementary file 2 — Supplemental Material [file 44172_2024_199_MOESM2_ESM.pdf]

Supplementary Information

Electrocaloric cooling with latent heat transfer enabling high power density

Authors: Julius Metzdorf[1]*, Patrick Corhan[1], David Bach[1], Sakyo Hirose[2], Dirk Lellinger[3], Stefan Mönch[4], Frank Kühnemann[1], Olaf Schäfer-Welsen[1], Kilian Bartholomé[1*]

Affiliations:

[1]Fraunhofer Institute for Physical Measurement Techniques IPM; Freiburg, 79110, Germany.

[2]Murata Manufacturing Co., Ltd., 1-10-1, Higashikotari, Nagaokakyo; Kyoto 617-8555, Japan.

[3]Fraunhofer Institute for Structural Durability and System Reliability LBF; 64289 Darmstadt, Germany.

[4]Fraunhofer Institute for Applied Solid State Physics IAF; Freiburg, 79108, Germany.

*Corresponding author. Email: Julius.metzdorf@ipm.fraunhofer.de

*Corresponding author. Email: Kilian.Bartholome@ipm.fraunhofer.de

The PDF file includes:

Supplementary Notes
Supplementary Figures 1 to 8
Supplementary Table 1

Other Supplementary Materials for this manuscript include the following:

Supplementary Video 1

Supplementary Notes

Thermal stabilization of EC-components

The AEH system needs a thermal stabilization mechanism, otherwise the temperature of the EC-components is slowly increasing, which leads to a failure of the heat transfer mechanism.

In any thermodynamic cycle, the amount of heat that is released to the hot side $\dot{Q}_h$ is always larger than the amount of heat that is taken up from the cold side $\dot{Q}_c$, i. e.:

$$\dot{Q}_h > \dot{Q}_c \tag{1}$$

This effect is even more enhanced, when the electrocaloric material shows dissipative effects like e.g. hysteretic behavior.

At the same time, in an electrocaloric heatpipe without local fluid return, the fluid entering the EC-segment from the evaporator $\dot{m}_c$ is in steady state always equal to the amount of fluid leaving the segment to the condenser $\dot{m}_h$ (see Supplementary Figure 1 left), i.e.:

$$\dot{m}_h = \dot{m}_c \tag{2}$$

Now, since in a heat pipe, the relevant heat transfer mechanism is condensation and evaporation of fluid, the amount of heat $\dot{Q}$ transferred by this process is proportional to the mass flow $\dot{m}$ and the enthalpy of evaporation Δh_V:

$$\dot{Q} = \dot{m}\, \Delta h_V \tag{3}$$

Thus, equations (5)- (7) cannot be fulfilled, which means, the system does not reach a steady state. Furthermore the enthalpy of evaporation is temperature-dependent $\Delta h_V = \Delta h_V(T) = \Delta h_V^T$ and is monotonically decreasing with rising temperature. The hotter a liquid is, the less energy is needed to evaporate it: $\Delta h_V^{T_h} < \Delta h_V^{T_c}$. This enhances the imbalance of the fluid and heat flux in the system even more.

In order to compensate for this imbalance, a local fluid return is implemented in the system.

Local fluid return: Concept and Implementation

The concept of the local fluid return is to transport some extra liquid fluid m_{FR} on the surface of the EC-components, so that the extra heat from the dissipative losses can be transferred to the fluid. Like this the EC-components are thermally stabilized and all heat during the heating can be expelled:

$$\dot{Q}_h = \dot{m}_h \, \Delta h_V = \Delta h_V (\dot{m}_c + \dot{m}_{FR}) \tag{4}$$

The local fluid return transports some extra fluid to the segment to balance the mass and heat flows, like schematically shown in Supplementary Figure 1 and Supplementary Figure 2.

The EC-components have a hydrophilic surface coating, which improves the wetting properties. For a homogenous thermal stabilization, the local fluid return is attached to both sides of the components (see Supplementary Figure 4).

Experimental proof of principle for local fluid return

In Supplementary Figure 3 the temporal evolution of a segment without thermal stabilization is compared to a segment thermally stabilized by a local fluid return.

In a system without thermal stabilization, to build up a temperature difference, the electric field strength was raised slowly over several minutes, so that the EC-components could fully exchange heat with the environment (see inset of Supplementary Figure 3).
Then the field was turned off, the EC-components cooled down below the temperature of the evaporator and fluid condensed on the EC-components. Now a sinusoidal field was applied to run the electrocaloric cooling cycle. At the beginning of cycling the condensed liquid is sufficient to transfer the heat from the EC-components. As dissipative losses slowly heat up the EC-components, each cycle a little bit more fluid evaporates than condenses. This leads to a dry-out of the EC-components and an interruption of heat transfer, which leads to a breakdown of the established temperature difference.
The performance of the segment with thermal stabilization is stable over time. More time is needed to establish a temperature difference. This is because more liquid fluid and thereby more thermal mass is present in the segment. The aspect, that the system with thermal stabilization leads to slightly smaller temperature spans can be explained by the additional fluid in the segment which corresponds to a larger thermal mass.

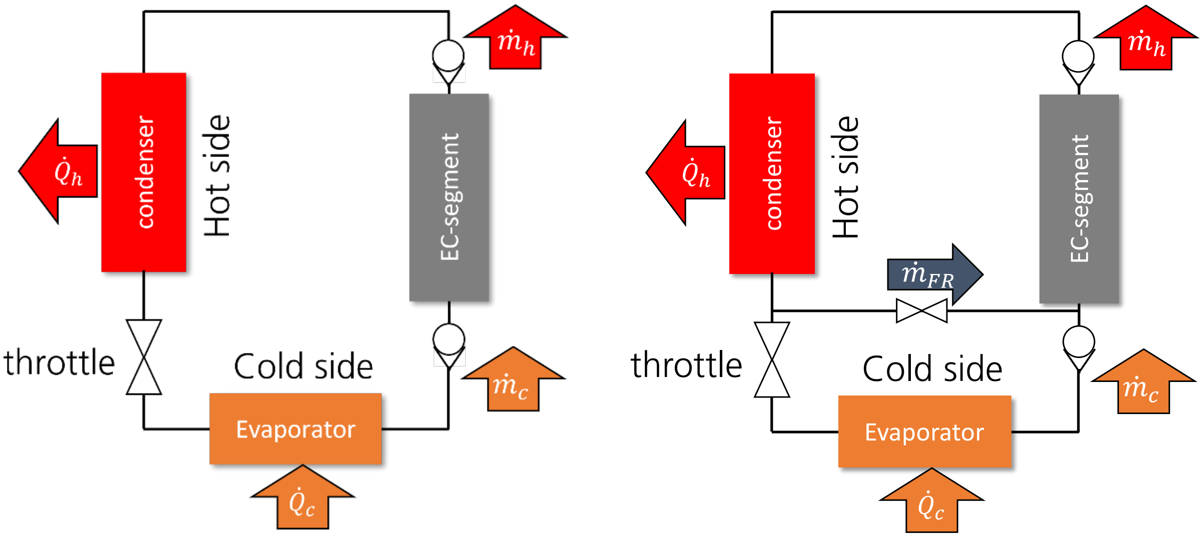

Supplementary Figure 1.
Left: The heat and mass flows of the active electrocaloric heatpipe without local fluid return. Fluid, that enters the EC-segment from the evaporator is transferred to the hot side. In a steady state, these fluid flows have to be equal, i.e. $\dot{m}_c = \dot{m}_h$.
Right: With a wick connecting the condenser to the EC-segment, fluid in the liquid state can passively be transferred to the EC-components. This local fluid return (FR) to the segment balances the mass flows and thermally stabilizes the EC-components.

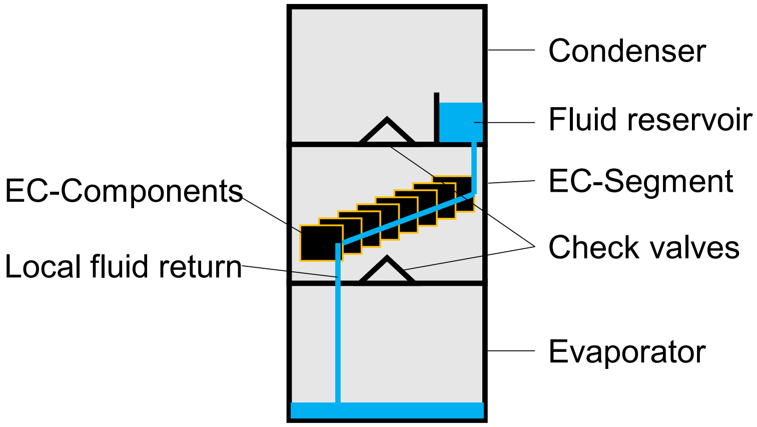

Supplementary Figure 2.

Simple illustration of the setup with local fluid return. If an alternating field is applied to the electrocaloric (EC) components, heat and fluid is transported from the evaporator through the EC-segment to the condenser. The fluid reservoir feeds the local fluid return, which stabilizes the EC-components.

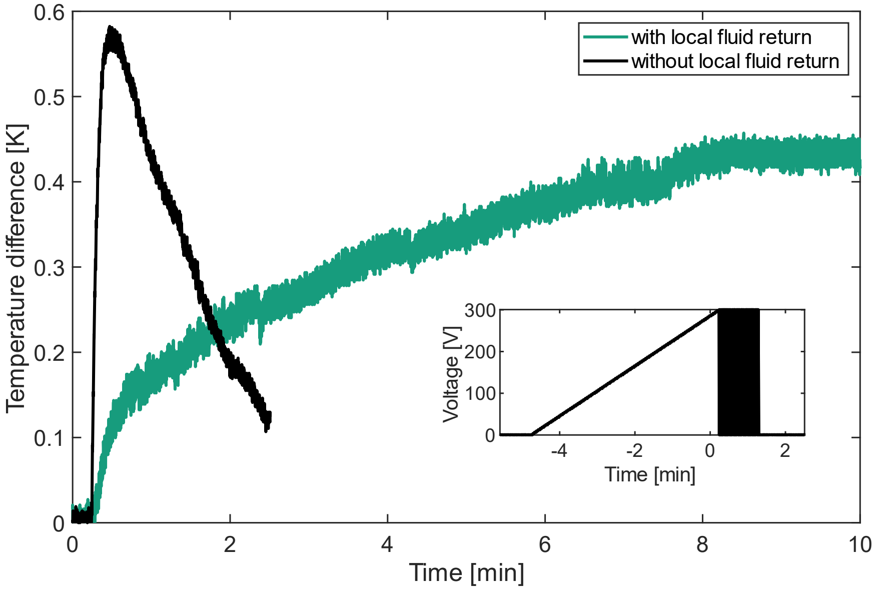

Supplementary Figure 3.

Temporal evolution of temperature difference of a segment without thermal stabilization (black line) and a segment with thermal stabilization (green line) provided by a local fluid return. To establish a temperature difference without local fluid return, the voltage was increased slowly over 5 min, so that the electrocaloric components could fully thermalize with the environment (see inset). Then the voltage is removed, so that liquid condenses on the EC-components. Then a sinusoidal voltage is applied, and a temperature difference can be established for a short time. Then the electrocaloric components heat up and the temperature difference decreases despite the applied voltage. After roughly 2.5 min the measurement was stopped. The green curve is the temperature difference established by a segment with local fluid return. More time is needed to establish a temperature difference, because more fluid and thus more thermal mass is present in the segment. The segment with local fluid return reaches a steady state.

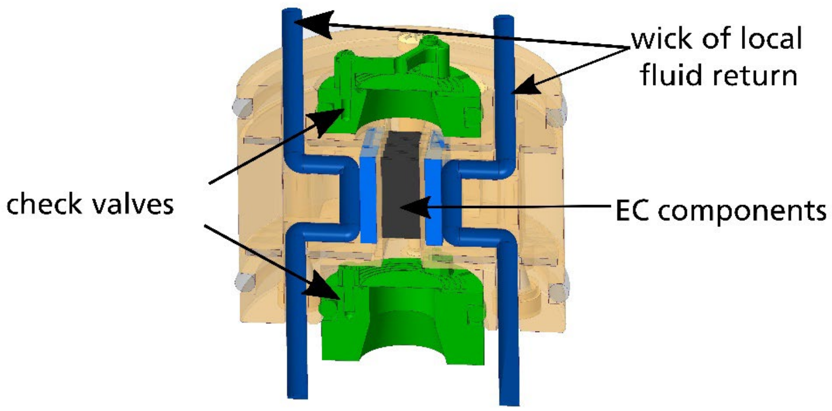

Supplementary Figure 4.

Schematic cross-section of the segment. The check valves (green), the electrocaloric (EC) components (dark grey) and the local fluid return (blue) are highlighted.

For a homogenous thermal stabilization, the local fluid return is attached to both sides of the components.

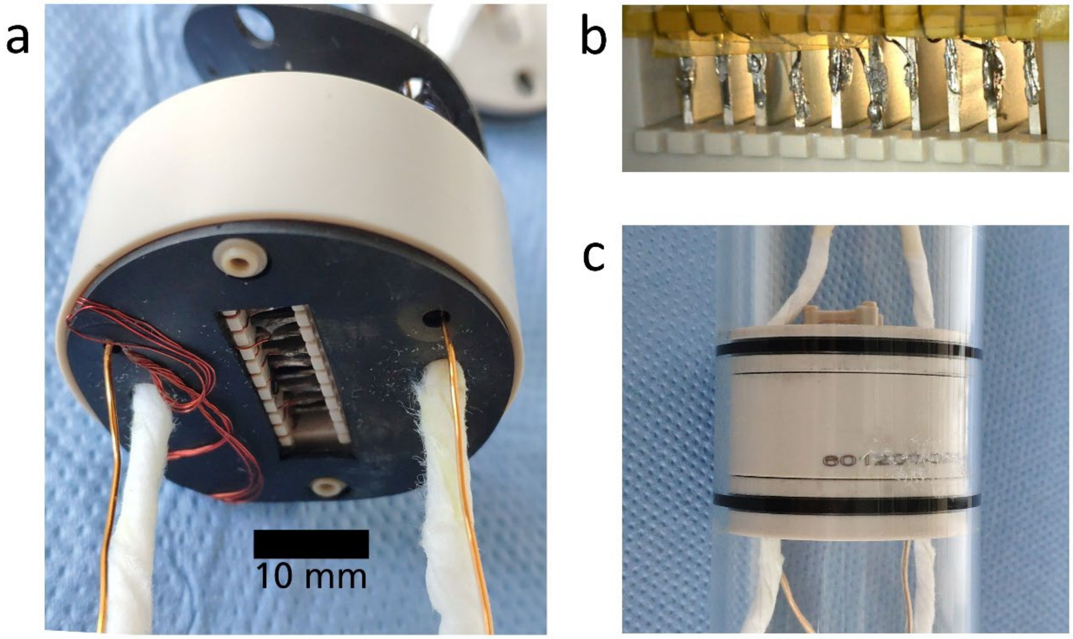

Supplementary Figure 5.
Experimental realization of the segment. (a) shows the half-open segment. Lead scandium tantalate (PST) components are in the middle, each electrically contacted with a thin wire with red protective coating. The thin wires are soldered to the thicker left copper wire. The right copper wire is connected to the other side of the PST-components. The wick of the local fluid return (white) can be seen on both sides. (b) shows the PST multi-layer-capacitors, which are placed in slits in the housing made of Polyether ether ketone (PEEK). (c) shows the finished segment within the glass tube as it is used in the setup. On the upper side part of the check valve can be seen.

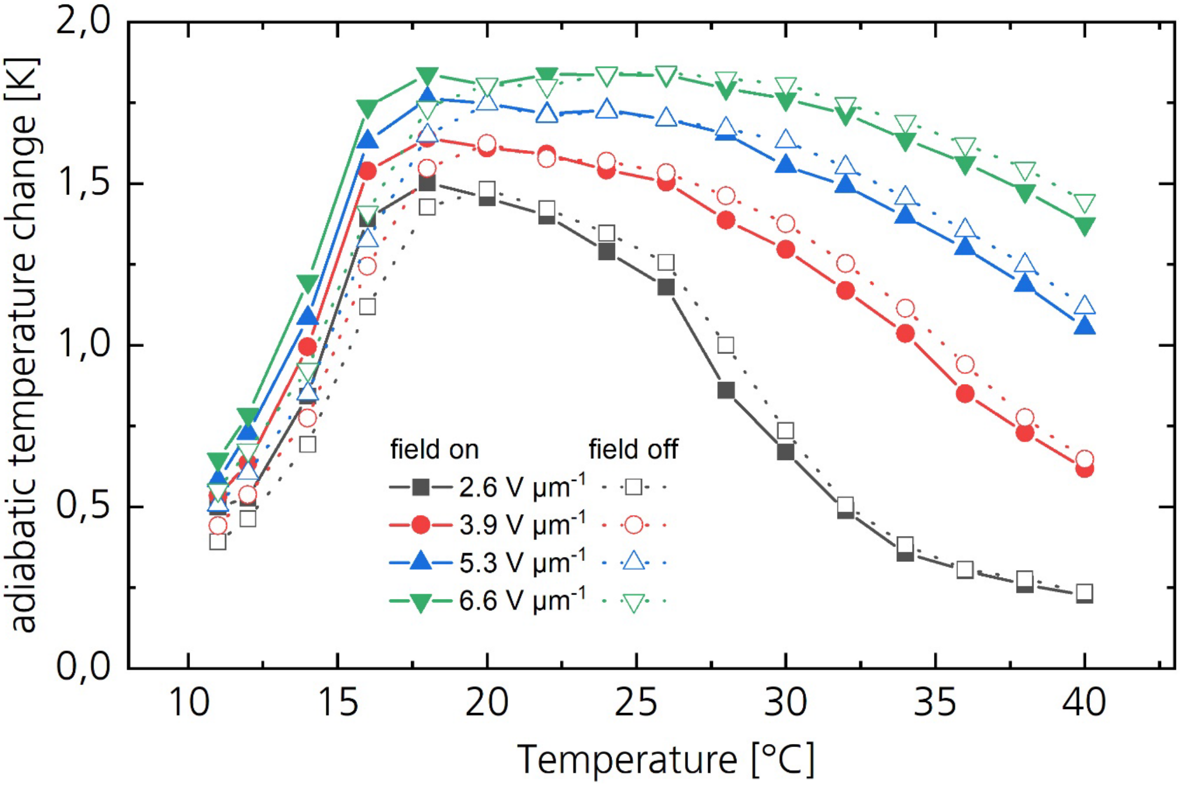

Supplementary Figure 6.
The temperature change on the surface of the components, when applying different fields was measured in air for a range of temperatures. The adiabatic temperature change was measured with a thermocouple. The temperature was set with a chiller, connected to a heat exchange plate in the same container as the electrocaloric components.

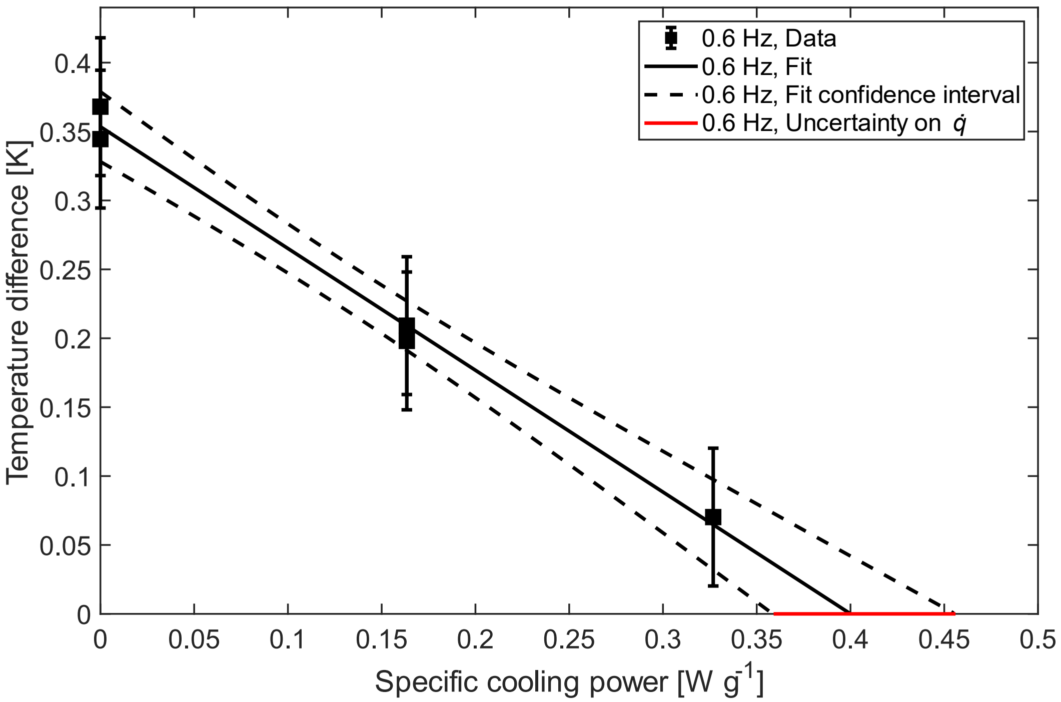

Supplementary Figure 7.
Uncertainty of specific cooling power. The uncertainty of the specific cooling power $\dot{q}(\Delta T = 0)$ is calculated from the interception of the lower and upper 95 % confidence interval of the linear regression with the x-axis ($\Delta T = 0$). For higher frequencies the slope is lower, therefore the uncertainty is higher.

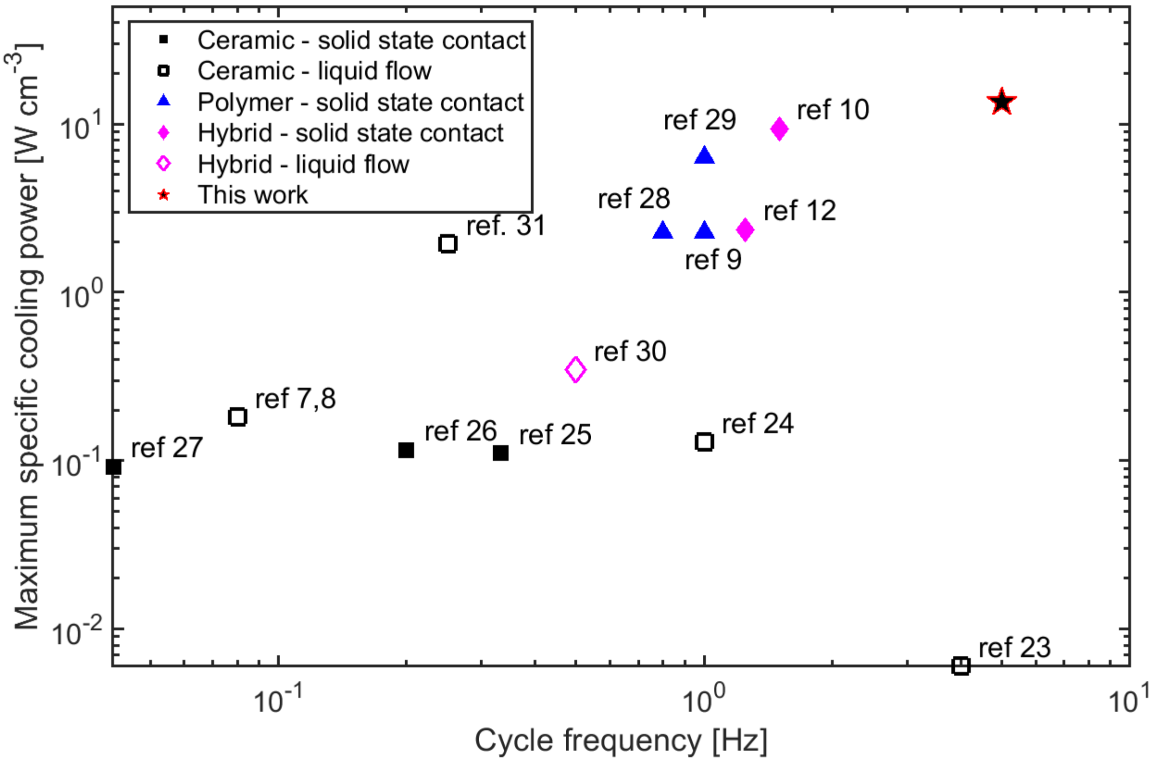

Supplementary Figure 8.
Maximum specific cooling power per active volume of electrocaloric prototype operated at their optimum cycle frequency. The empty symbols refer to all prototype, which use fluids for the heat transport, the filled symbols refer to all prototype which use solid state contact for heat transfer. This work, which uses evaporation and condensation as a heat transfer mechanism is shown as a star.

Supplementary Table 1.

Comparison of cooling powers of different prototypes. Used ceramic EC-materials are $PbSc_{0.5}Ta_{0.5}O_3$ (PST), (1-x)$Pb(Mg_{1/3}Nb_{2/3})O_3$–x$PbTiO_3$ (PMN-xPT), $PbZr_{0.95}Ti_{0.05}O_3$ (PZT) or $BaTiO_3$ (BTO). They are either bulk material or multilayer capacitors (MLCs). Polymer EC-material is based on polyvinylidene difluoride (PVDF). Hybrid materials used $Ba_{0.6}Sr_{0.4}TiO_3$ (BST) or $Ba_xSr_{1-x}TiO_3$ (BSTO) nanoparticles (NP) or a $Ba_{0.85}Ca_{0.15}Zr_{0.1}Ti_{0.9}O_3$ (BCZT) network within PVDF.

Reference	Heat transfer mechanism	EC-material	m_{active} [g]	Frequency [Hz]	$\dot{q}$ [W g^{-1}]	$\dot{q}$ [W cm^{-3}]	$\dot{Q}$ [W]	ΔT [K]
[23]	Fluid-convection	PST-bulk	14 [a)]	4	0.0007	0.006	0.01	2.5
[24]	Fluid-convection	PMN-bulk	11.7	1	0.02	0.13	0.23	3.1
[7,8]	Fluid-convection	PST MLCs	11.4 [b)]	0,08	0.02	0.181	0.23	13.0
[25]	Solid state contact	BTO MLCs	0.87	0.33	0.041	0.111	0.036	0.3
[26]	Solid state contact	PST MLCs	6.4 [c)]	0.2	0.013	0.116	0.085	5.2
[27]	Solid state contact	BTO MLCs	1.09	0.04	0.015	0.092	0.017	0.26
[28]	Solid state contact	PVDF MLCs	0.105	0.8	2.83	2.260	0.30	2.8
[9]	Solid state contact	PVDF MLCs	0.7	1	1.29	2.265	0.91	8.7
[29]	Solid state contact	PVDF MLCs	0.196	1	3.6	6.300	0.34	4.8
[10]	Solid state contact	PVDF MLCs with BST NP	0.060 [d)]	1.5	3.95	9.390	0.24	4.6
[12]	Solid state contact	BCZT network in PVDF	0.156 [e)]	1.25	1.13	2.360	0.17	--
[30]	Fluid-convection	PVDF with BSTO NP	--	0.5	0.35	0.702[f)]	--	1.4
[31]	Fluid-convection	PST MLCs	26.16	0.25	0.214	1.98	5.6	9
This work	Evaporation and condensation	PST MLCs	1.53	5	1.48	13.47[g)]	2.2	0.4

a) Not stated, whether it is active or total mass.
b) It was assumed that 60% of the mass were active as stated in [8].
c) Active volume was calculated by dividing absolute cooling power by volumetric cooling power. Mass was calculated by multiplying volume with density (given in the supplementary information).
d) Active volume of all 9 pixels is calculated to be 25.1 mm^3. Density is 2.38 g cm^{-3} (given in the supplementary information of the publication).
e) Active volume is 75 mm^3. Density was given by authors on request with 2.08 g cm^{-3}.
f) Almost all the volume/mass is active. This should be considered, when comparing sizes/masses of prototype.
g) Using a PST density of 9.07 g cm^{-3} [21].

Supplementary Video 1

Working principle of the active electrocaloric heatpipe.
