# Supplementary material for: Electrocaloric cooling system utilizing latent heat transfer for high power density
Source: Commun Eng. 2024 Mar 21;3:55. doi: 10.1038/s44172-024-00199-z (PMC10957880; doi:10.1038/s44172-024-00199-z)
Supplement: Supplementary file 3 — Description of Additional Supplementary Files [file 44172_2024_199_MOESM3_ESM.pdf]

Description of Additional Supplementary Files

File name: Supplementary Video 1

Description: Working principle of the active electro caloric heat pipe.
